# Regional differences in cervical cancer incidence and associated risk behaviors among Norwegian women: a population-based study

**DOI:** 10.1186/s12885-021-08614-w

**Published:** 2021-08-19

**Authors:** Bo T. Hansen, Suzanne Campbell, Mari Nygård

**Affiliations:** Department of Research, Cancer Registry of Norway, Postbox 5313 Majorstuen, 0304 Oslo, Norway

**Keywords:** HPV, Cervical cancer, Epidemiology, Incidence, Cervical screening, Sexual behavior, Social norms, Risk factors

## Abstract

**Background:**

Cervical cancer incidence is influenced by screening and risk factors in the population. The main risk factor for cervical cancer is sexually transmitted human papillomavirus (HPV), which is sexually transmitted and thus associated with sexual behavior. Smoking, parity and hormonal contraceptive use may also be associated with cervical cancer risk. We compared incidence, screening coverage and risk behaviors for cervical cancer between health regions in Norway.

**Methods:**

We obtained data on incidence of cervical cancer among Norwegian women during 1992–2016 and data on screening coverage from the Cancer Registry of Norway. We obtained data on sexual behavior and smoking from a population-based survey of 16,575 Norwegian women who were 18–45 years old in 2005.

**Results:**

Cervical cancer incidence was higher in the northern and southeastern region than in the middle and western region (range in incidence per 100,000 person-years during 1992–2016; north: 10.5 to 14.6; southeast: 9.3 to 12.9; mid: 6.8 to 9.5; west: 8.4 to 10.0). The incidence decreased modestly in the north (average annual percentage change (95% confidence interval) − 1.0 (− 1.2 to − 0.7)) and southeast (− 0.7 (− 1.0 to − 0.3)), but did not change significantly in the mid (− 0.3 (− 1.0 to 0.4)) and west (− 0.3 (− 0.6 to 0.0)). Compared to the national average, women in the north had earlier sexual debut, more partners and higher prevalence of ever having had a sexually transmitted infection (STI), while the opposite was observed among women in the west. Women in the middle and southeastern regions tended to be similar to the national average for sexual behaviors. Although less pronounced, the prevalence of smoking showed regional patterns similar to that observed for sexual behaviors, while ever-use of hormonal contraceptives and cervical screening coverage was similar between regions.

**Conclusions:**

There were regional differences in cervical cancer incidence during the era of nationally organized cervical screening in Norway. To some extent, these differences corresponded to regional differences in risk behavior for cervical cancer in the Norwegian female population.

**Supplementary Information:**

The online version contains supplementary material available at 10.1186/s12885-021-08614-w.

## Introduction

Screening has reduced the incidence of cervical cancer substantially in many countries [1], including Norway where a reduction of approximately 70% has been estimated [2]. Cervical cancer still represents a considerable health burden because many women do not attend screening as recommended, screening does not detect all pre-invasive lesions and treatment of pre-invasive lesions is not always successful [3]. Women who do not attend screening as recommended have a higher risk of cervical cancer and have cancer diagnosed at a more advanced stage [4]. Worldwide, cervical cancer ranks as the fourth most frequently diagnosed cancer among women [5]. In Norway, which has had widespread screening since the early 70s, it is no longer among the top 10 most frequently diagnosed cancers among women overall, but still ranks as the third most common cancer among women age 25–49. The current standardized incidence rate of cervical cancer in Norway is 10.7 per 100,000 person-years, and the median age at cervical cancer diagnosis is 45 years [6].

Human papillomavirus (HPV) is considered a necessary cause for invasive cervical cancer [7]. HPV is sexually transmitted, thus the risk of exposure to the virus is strongly associated with sexual behavior such as the age at first intercourse and the number of sexual partners accrued [8]. Other risk factors include smoking [9], oral contraceptive use [10] and parity [11], all of which to some extent also may be associated with sexual behavior. HPV infection is very common, but although most women who are sexually active will be exposed to HPV during their lifetime, only a small fraction will develop a persistent infection by an oncogenic HPV type that progresses to a pre-invasive lesion, which may further progress to cancer [12]. HPV vaccines have recently been introduced, but due to the long duration between HPV exposure and cancer development, HPV vaccination has not yet impacted substantially on cervical cancer incidence at the population level.

The risk of cervical cancer is influenced by attendance to screening, the quality of the screening-associated health services and the exposure to risk factors for cervical cancer. All these factors may differ between countries due to differences in screening practices and social norms. Recently, some countries with organized screening programs against cervical cancer also report differences in cervical cancer incidence between regions within countries [13–15]. In the present study, we address whether there are regional differences in cervical cancer incidence, screening coverage and risk behaviors for cervical cancer in Norway during the era of nationally organized cervical screening.

## Methods

### Registry data

Cancer data was extracted from the Cancer Registry of Norway (CRN). The CRN receives data from clinicians, pathology laboratories and the Cause of Death Registry, and notification of cancer diagnoses to the CRN is compulsory by law, which ensures high data completeness and quality [16]. For the period 1992–2016, we extracted data on all incident primary cases of malignant cervical cancer (ICD-10 topography code C53). For each case, we extracted the date of diagnosis, patient age at diagnosis, tumor topography and morphology. Age-specific population size by calendar year was obtained from the National Registry, which contains individual core demographic data on all Norwegian citizens. The CRN is also responsible for the Norwegian Cervical Cancer Screening Program (NCCSP), which was launched in 1995, following piloting since 1992 and extensive opportunistic screening since the 70s. The NCCSP individually reminds women aged 25–69 who have not had a cervical smear as recommended to make an appointment for smear-taking. Cytological screening every third year was the program recommendation during the study period. Screening coverage data was obtained from the NCCSP. All registry data analyses were performed with anonymous data.

### Survey data

Details about the survey have been published previously [17]. In brief, a self-administrated questionnaire on lifestyle and health was mailed to 25,001 Norwegian women aged 18–45 (born 1959–1986) during November 2004–June 2005. The women were randomly sampled from the National registry. In total, 24,424 women were reached, of which 16,575 answered the questionnaire (via paper, web or phone), giving a total survey response rate of 68%. Only authorized personnel at the Cancer Registry of Norway had access to the identity of the women invited to participate in the survey. All survey data analyses were performed with de-identified data.

### Statistics

We analyzed cervical cancer incidence and behavior associated with cervical cancer risk by the four administrative health regions in Norway. Overall, the distribution by broad age, education and immigration categories was similar between women living in each of the regions in 2005, although some differences were observed. Compared to the other regions, the Southeast had a higher proportion of immigrant women, while the North had a higher proportion of women with primary education and slightly lower proportions of women with secondary and university education (Supplementary Table 1).

We present incidence rates per 100,000 woman-years, age-standardized by the World Standard Population [18]. Temporal incidence trends by age-standardized yearly rates were assessed by joinpoint regression. The analysis identifies segments with distinct linear slopes that can be connected by joinpoints, and determines how many (if any) joinpoints should be used to best describe trends in the data [19]. The minimum and maximum number of joinpoints allowed in the time series were zero and four, respectively, and there had to be at least four data points between any joinpoints and between a joinpoint to either end of the data series. The annual percentage change (APC) with 95% confidence interval (CI) was estimated for each segment by fitting a linear regression to the logarithm of the rates by calendar year. We also estimated the average annual percentage change (AAPC) over the whole period 1992–2016. For data series that include joinpoints, the AAPC is the average of the individual APCs weighted by the length of each segment [20]. Significance tests of joinpoint regressions were performed by Monte Carlo permutation with 4499 replicates.

We limited analyses of age at first intercourse to integer ages that all surveyed women had experienced, i.e. up to and including age 17. We used survival models to estimate hazard ratios (HRs) with corresponding 95% CIs to compare rates of first intercourse by region, with age as the timescale. Participants were followed from birth until age at first intercourse if they had first intercourse before age 18, or they were censored when turning 18. Since age at first intercourse was reported as an integer, we used a discrete-time hazard model with a complementary log-log link function [21]. We additionally analyzed first intercourse before legal age of consent in Norway (i.e. before age 16) as a dichotomous outcome in a logistic regression.

Since the distribution of number of sexual partners is highly right-skewed, we categorized the number of partners into four ordered categories (1–2, 3–5, 6–10 and > 10 partners), and analyzed it by generalized ordinal regression in a cumulative logit model [22]. The cutpoints were defined by the national quartile distribution of number of partners among women who had debuted sexually (2, 5 and 10 partners for q1, q2 and q3, respectively). At each cutpoint, the model provides odds ratios (ORs) of having more partners, for each region compared to the reference level. We analyzed ever having had a sexually transmitted infection (STI; here defined as a combined endpoint of ever having had at least one of the following: chlamydia, gonorrhea, herpes, trichomonas vaginalis, genital warts), current smoking and ever having used hormonal contraceptives as separate dichotomous outcomes by logistic regression. Women with missing answers to specific questionnaire items were excluded from analyses of that item, thus sample size varies between models. Since no region is an obvious reference relative to the other regions, survey data models were performed with sum contrasts [23], i.e. with the national level as the reference against which each region is compared. Survey data plots were loess smoothed with bandwidth 0.3. All survey models were adjusted for age and mode of response. The model on number of sexual partners was in addition adjusted for age at sexual debut, and the models on ever having had an STI and ever having used hormonal contraceptives were in addition adjusted for age at sexual debut and number of sexual partners. Statistical computing was performed in R [24, 25] and Joinpoint [19]. Two-sided *P*-values were considered significant when they were < 0.05.

## Results

### Regional incidence of cervical cancer

The incidence rate of cervical cancer per 100,000 women-years was generally somewhat higher in the northern region than in the other regions during 1992–2016, ranging from 10.5 to 14.6 among the five-year subperiods (Table 1). The southeastern region also had a consistently higher incidence rate than the middle and western regions, ranging from 9.3 to 12.9 during the subperiods. The corresponding incidence rate ranges for cervical cancer in the western and middle regions were 8.4 to 10.0 and 6.8 to 9.5, respectively (Table 1). A similar pattern was observed for squamous cell cancers, for which the incidence rate ranged from 7.2 to 12.3 in the north, 6.8 to 9.9 in the southeast, 6.3 to 7.6 in the west and 4.3 to 7.8 in the middle region, during 1992–2016. For the rarer adenocarcinomas, the regional differences were less pronounced. The highest incidence rates were observed in the north and in the southeast (ranging from 1.4 to 2.6, and from 1.6 to 2.5, respectively), while somewhat lower rates were observed in the western and middle regions (ranging from 1.6 to 2.0, and from 1.1 to 1.8, respectively) (Table 1).

**Table 1:** Age-standardized incidence rate of cervical cancer per 100.000 women-years (95% CI^1^) by region, period and histology in Norway

Histology/ period	Region north			Region middle			Region southeast			Region west			% regional difference^**2**^
	N	Incidence (95% CI)		N	Incidence (95% CI)		N	Incidence (95% CI)		N	Incidence (95% CI)		
All cancer													
1992–1996	191	12.7	(10.9, 14.7)	200	9.5	(8.1, 11.0)	1042	12.9	(12.1, 13.8)	275	10.0	(8.8, 11.4)	35.8
1997–2001	217	14.6	(12.7, 16.8)	165	7.8	(6.6, 9.2)	905	10.7	(10.0, 11.4)	254	8.8	(7.7, 10.1)	87.2
2002–2006	185	12.4	(10.6, 14.5)	148	6.8	(5.7, 8.1)	858	9.8	(9.1, 10.5)	289	9.4	(8.3, 10.7)	82.4
2007–2011	170	12.3	(10.4, 14.4)	178	8.5	(7.2, 9.9)	844	9.3	(8.7, 10.0)	264	8.4	(7.4, 9.6)	46.4
2012–2016	155	10.5	(8.8, 12.4)	206	9.5	(8.2, 11.0)	999	10.8	(10.1, 11.5)	303	9.3	(8.2, 10.4)	16.1
SCC^3^													
1992–1996	154	10.4	(8.7, 12.2)	162	7.8	(6.5, 9.2)	793	9.9	(9.1, 10.6)	203	7.6	(6.6, 8.8)	36.8
1997–2001	183	12.3	(10.5, 14.3)	123	5.8	(4.8, 7.0)	708	8.5	(7.8, 9.2)	199	6.9	(5.9, 7.9)	112.1
2002–2006	144	9.7	(8.1, 11.5)	97	4.3	(3.5, 5.4)	629	7.0	(6.4, 7.6)	208	6.8	(5.9, 7.8)	125.6
2007–2011	128	9.4	(7.7, 11.3)	136	6.6	(5.5, 7.9)	622	6.8	(6.3, 7.4)	194	6.3	(5.4, 7.3)	49.2
2012–2016	105	7.2	(5.8, 8.8)	158	7.3	(6.1, 8.6)	712	7.7	(7.1, 8.3)	226	6.9	(6.0, 7.9)	11.6
ADC^4^													
1992–1996	20	1.4	(0.8, 2.2)	22	1.1	(0.7, 1.7)	161	2.0	(1.7, 2.4)	53	1.8	(1.3, 2.4)	81.8
1997–2001	24	1.7	(1.1, 2.6)	32	1.6	(1.1, 2.3)	133	1.6	(1.3, 1.9)	43	1.6	(1.2, 2.2)	6.3
2002–2006	28	1.9	(1.2, 2.8)	32	1.6	(1.1, 2.4)	169	2.0	(1.7, 2.4)	58	2.0	(1.5, 2.6)	25.0
2007–2011	31	2.3	(1.6, 3.4)	33	1.6	(1.1, 2.3)	164	1.9	(1.6, 2.2)	52	1.7	(1.2, 2.2)	43.8
2012–2016	38	2.6	(1.8, 3.6)	36	1.8	(1.2, 2.5)	222	2.5	(2.1, 2.8)	57	1.8	(1.3, 2.3)	44.4

^1^ Confidence interval

^2^ Highest vs. lowest regional incidence

^3^ Squamous cell cancer

^4^ Adenocarcinoma

The regional differences in cervical cancer incidence rates were particularly high during 1997–2001 and 2002–2006, and they were driven by differences in the squamous cell carcinoma incidence. During these 5-year periods, the incidence rate of squamous cell carcinoma was more than twice as high in the region with the highest incidence (the north) compared to the region with the lowest incidence (the middle). The differences between regions in the five-yearly incidence rates decreased after 2006 for all cervical cancer combined and for squamous cell cancer, but not for adenocarcinoma (Table 1).

**Fig. 1:**
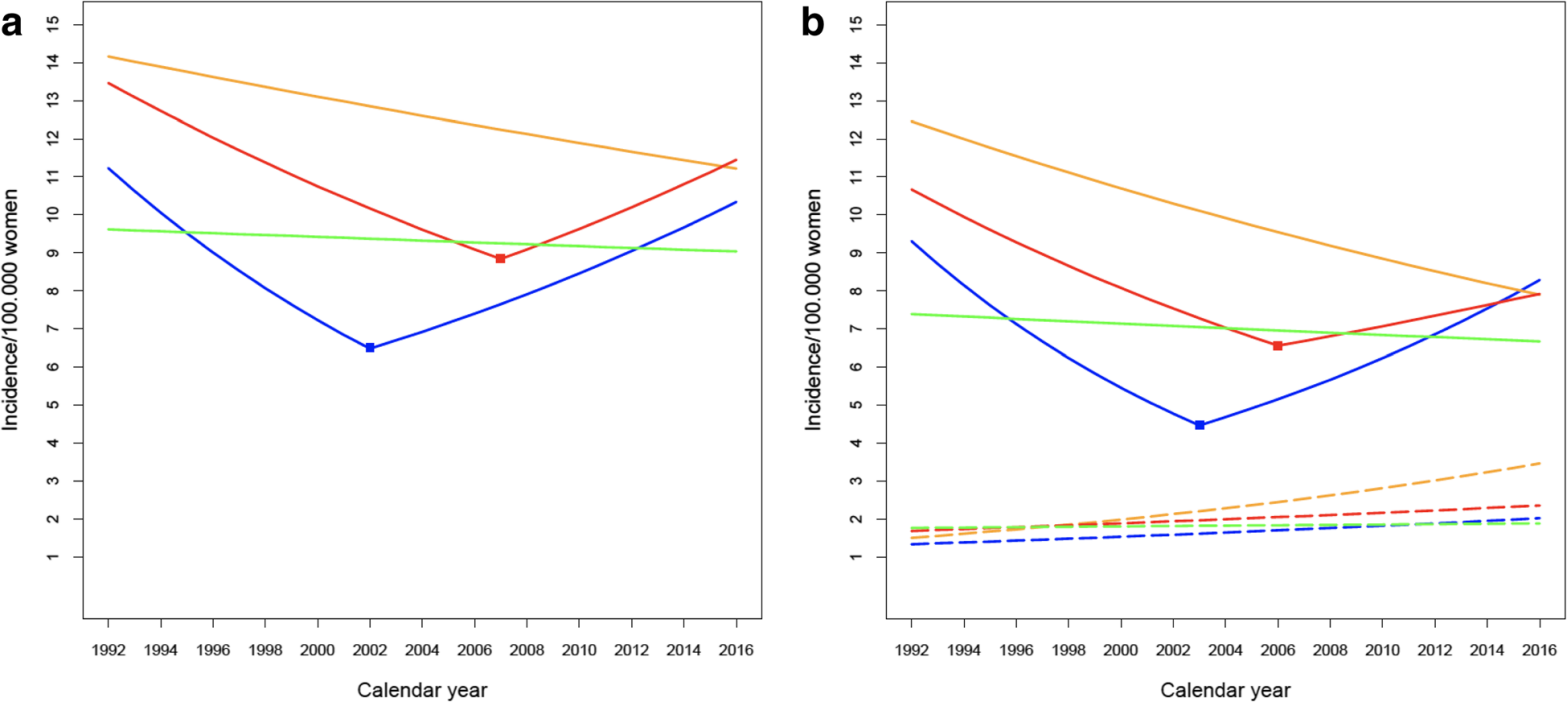
Age standardized incidence trends per 100,000 women-years for cervical cancer in Norway 1992–2016 by region (north = yellow, middle = blue, southeast = red, west = green). **A**) All cervical cancer combined. **B**) Squamous cell carcinoma (solid lines) and adenocarcinoma (dashed lines). Shown are the best-fitting trend line for each region and the joinpoints (if any) from joinpoint regression analyses

Modest changes in yearly cervical cancer incidence trends were observed for some of the regions during the period investigated. In the northern region, the overall cervical cancer incidence trend for the entire 1992–2016 period was decreasing, with an AAPC of − 1.0 (95% CI − 1.2 to − 0.7), and without changes in trend observed over the period (Table 2, Fig. 1A). The negative incidence trend in the north was mostly explained by a consistent reduction in squamous cell cancer (AAPC (95% CI): − 1.9 (− 2.3 to − 1.5)). In contrast, the adenocarcinoma incidence in the northern region steadily increased during the same period and had the largest AAPC observed in the current study (3.5 (2.7 to 4.4)), corresponding to nearly a doubling of the number of adenocarcinoma cases in this region over the study period (Table 1). The southeastern region also had a slight decrease in the overall cervical cancer rate over the study period (AAPC -0.7 (− 1.0 to − 0.3)). However, there was a trend shift in the southeast in 2007, with a decrease during 1992–2007 and an increase during 2007–2016 (Table 2, Fig. 1A). The same trend could be observed for SCC, with a decreasing AAPC for the whole period (− 1.2 (− 1.6 to − 0.9)), but with two opposing trends during the study period (Table 2, Fig. 1B). Adenocarcinomas increased steadily in the southeastern region during 1992–2016 (Table 2, Fig. 1B), with an AAPC of 1.4 (1.0 to 1.8). The middle region showed patterns similar to the southeastern region, with a decreasing trend for the overall cervical cancer and SCC during the first decade, followed by an increasing incidence trend (Fig. 1A, B, Table 2). The AAPC did not change significantly in the middle region, neither for overall cervical cancer (− 0.3 (− 1.0 to 0.4)) nor for SCC (− 0.5 (− 1.4 to 0.4)), while the adenocarcinoma incidence showed a steady increase over the study period (AAPC 1.7 (1.1 to 2.4)). The western region showed little change in cervical cancer incidence during 1992–2016 (Fig. 1A, B), with non-significant AAPCs for overall cervical cancer (− 0.3 (− 0.6 to 0.0)) and adenocarcinoma (0.3 (− 0.3 to 0.9)), a slight decrease in SCC (− 0.4 (− 0.8 to − 0.1)), and no trend change over the study period (Table 2).

**Table 2:** Cervical cancer incidence trend analyses by region and histology in Norway during 1992–2016

Histology	Region	AAPC^**1**^ (95% CI^**2**^)	Joinpoint^**3**^	Subperiod	APC^**4**^ (95% CI)
All cancer	North	-1.0 (−1.2, −0.7)*	None		
	Middle	−0.3 (−1.0, 0.4)	2002	1992–2002	−5.4 (−6.6, −4.1)*
	Middle			2002–2016	3.4 (2.6, 4.2)*
	Southeast	−0.7 (−1.0, − 0.3)*	2007	1992–2007	−2.8 (−3.1, −2.4)*
	Southeast			2007–2016	2.9 (2.1, 3.7)*
	West	−0.3 (− 0.6, 0.0)	None		
SCC^5^	North	−1.9 (−2.3, − 1.5)*	None		
	Middle	−0.5 (−1.4, 0.4)	2003	1992–2003	−6.4 (−7.8, −5.1)*
	Middle			2003–2016	4.9 (3.6, 6.1)*
	Southeast	−1.2 (−1.6, −0.9)*	2006	1992–2006	−3.4 (−3.8, − 3.1)*
	Southeast			2006–2016	1.9 (1.3, 2.6)*
	West	−0.4 (−0.8, − 0.1)*	None		
ADC^6^	North	3.5 (2.7, 4.4)*	None		
	Middle	1.7 (1.1, 2.4)*	None		
	Southeast	1.4 (1.0, 1.8)*	None		
	West	0.3 (−0.3, 0.9)	None		

^1^ Average annual percentage trend from 1992 to 2016

^2^ Confidence interval

^3^ Whether/when (calendar year) trend changed, from joinpoint regression analysis

^4^ Annual percentage change for subperiod with distinct incidence trend

^5^ Squamous cell cancer

^6^ Adenocarcinoma

* *P* < 0.05

### Sexual behavior, smoking and screening coverage by region

**Fig. 2:**
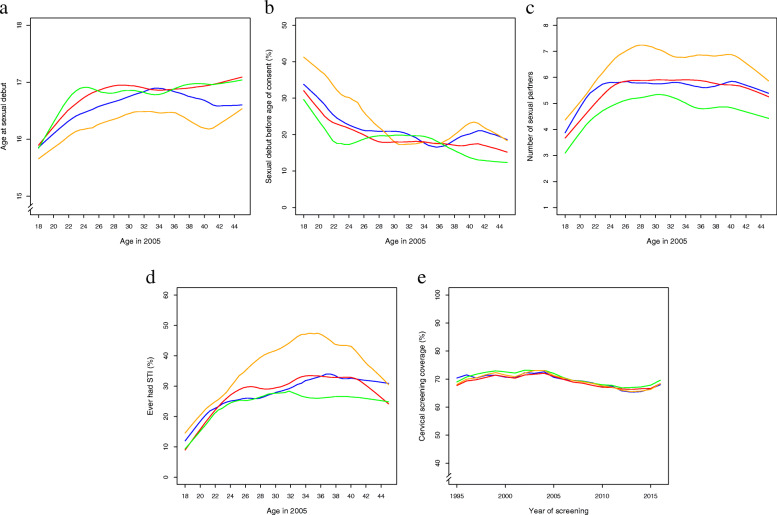
a-d: Loess smoothed estimates of sexual behavior by age and region (north = yellow line, middle = blue line, southeast = red line, west = green line), among Norwegian women aged 18–45 in 2005: **A**) Age at sexual debut; **B**) Sexual debut before legal age of consent, i.e. before age 16 (%); **C**) Number of sexual partners; **D**) Ever had an STI (combined % of ever had chlamydia, gonorrhea, herpes, trichomonas vaginalis and/or genital warts); and **E**) Cervical screening coverage rates by calendar year and region. Coverage is shown as the % of women eligible for screening in the Norwegian cervical screening program who had at least one screening test during the previous 3.5 years

For all regions, age at sexual debut increased somewhat with increasing age (Fig. 2A), while the frequency of debut before legal age of consent generally decreased with increasing age among Norwegian women surveyed at age 18–45 in 2005 (Fig. 2B). The age at sexual debut was generally lower in the northern region compared to the other regions, for all ages surveyed (Fig. 2A). This is reflected in the quartile estimates for age at sexual debut, which ranged 15–17 with median 16 in the north, compared to 16–18 with median 17 for all other regions, and in the adjusted hazard ratio for sexual debut for the north compared to the national reference level (HR (95% CI): 1.30 (1.24 to 1.36)) (Table 3). Moreover, the frequency of sexual debut before legal age of consent (Fig. 2B) was 30.9% in the northern region, which was 10 percentage points higher than in Norway as a whole, resulting in significantly higher adjusted odds for sexual debut before age of consent (OR (95% CI): 1.55 (1.42 to 1.70)). Compared to the national average, the middle region did not differ in adjusted hazard ratio of sexual debut (1.04 (0.99 to 1.09)), while the southeastern and western regions had a higher age at sexual debut with adjusted hazard ratios of 0.88 (0.85 to 0.91) and 0.85 (0.81 to 0.88), respectively. A concordant pattern was observed in the adjusted logistic regression of sexual debut before age of consent, with no difference for the middle region and significantly lower odds for the southeast and west, compared to the national average (Fig. 2B, Table 3).

**Table 3:** Sexual behavior by region in Norway

Age at sexual debut							
Region	N	Debut (%)	Debut before age of consent^1^ (%)	Mean (SE^2^) age at debut	Median (IQR^3^) age at debut	Adj HR^4^ (95% CI^5^) debut	Adj OR^6^ (95% CI) debut before age of consent
Norway	16,255	96.26	20.9	17.19 (0.02)	17 (16, 18)	1.00 (reference)	1.00 (reference)
North	1643	97.58	30.9	16.53 (0.06)	16 (15, 17)	1.30 (1.24, 1.36)*	1.55 (1.42, 1.70)*
Middle	1939	96.87	22.1	16.97 (0.06)	17 (16, 18)	1.04 (0.99, 1.09)	0.97 (0.89, 1.06)
Southeast	9351	96.36	19.8	17.27 (0.03)	17 (16, 18)	0.88 (0.85, 0.91)*	0.86 (0.81, 0.91)*
West	3322	94.93	18.4	17.39 (0.05)	17 (16, 18)	0.85 (0.81, 0.88)*	0.77 (0.71, 0.83)*
**Number of sexual partners**							
**Region**	**N**^7^	**Median (IQR)**	**Mean (SE)**	**Adj OR**^**8**^**(95% CI) > 2 partners**		**Adj OR**^**8**^**(95% CI) > 5 partners**	**Adj OR**^**8**^**(95% CI) > 10 partners**
Norway	15,517	5 (2, 10)	7.61 (0.08)	1.00 (reference)		1.00 (reference)	1.00 (reference)
North	1573	6 (3, 10)	8.80 (0.24)	1.26 (1.13, 1.40)*		1.17 (1.07 1.27)*	1.11 (1.01, 1.23)*
Middle	1867	5 (2, 10)	7.44 (0.18)	0.96 (0.88, 1.06)		0.97 (0.90, 1.05)	0.98 (0.89, 1.08)
Southeast	8945	5 (2, 10)	7.74 (0.11)	1.05 (0.99, 1.11)		1.05 (1.00, 1.11)	1.11 (1.04, 1.18)*
West	3130	5 (2, 8)	6.76 (0.16)	0.79 (0.73, 0.85)*		0.84 (0.79, 0.90)*	0.83 (0.76, 0.90)*

^1^ Debut before age 16

^2^ Standard error

^3^ Interquartile range

^4^ Hazard ratio adjusted for age and mode of response, from discrete-time generalized linear survival model with follow-up from birth until age 18

^5^ Confidence interval

^6^ Odds ratio adjusted for age and mode of response, from logistic regression

^7^ Number of women with sexual debut who reported number of sexual partners

^8^ Odds ratio adjusted for age, mode of response and age at sexual debut, from ordinal regression

* *P* < 0.05

In all regions, the average number of sexual partners increased with age at survey response up until the women were in their mid/late 20s, while the number of partners did not change or declined slightly among successively older women in the survey of Norwegian women aged 18–45 in 2005. Moreover, any regional differences observed were similar across the age range of the women surveyed (Fig. 2C). The quartile number of sexual partners reported at the national level ranged 2 to 10, with a median of 5 partners (Table 3). The quartiles observed for the national level were also observed in the middle region and southeastern regions, while they ranged 3 to 10 with a median of 6 partners in the northern region, and 2 to 8 with a median of 5 partners in the western region (Table 3). Consequently, compared to the national average, the adjusted likelihood of reporting a higher number of sexual partners was higher in the northern region at each of the cutpoints of the ordinal regression analysis, and lower in the western region, while it did not differ significantly from the national level in the middle region (Table 3). In the southeastern region, there was a tendency for higher adjusted odds for more partners, which was marginally nonsignificant at the two lowest cutpoints. At the highest cutpoint, the adjusted odds ratio for having had more than 10 sexual partners was 1.11 (1.01 to 1.23) in the north, 0.83 (0.76 to 0.90) in the west, 0.98 (0.89 to 1.08) in the middle, and 1.11 (1.04 to 1.18) in the southeastern region, relative to the national average.

The patterns observed for the number of sexual partners were echoed in the prevalence of ever having had an STI. The national prevalence among women aged 18–45 in 2005 was 27.8%. By region, it was 36.1% in the north, 27.3% in the middle, 27.8% in the southeast, and 23.9% in the west. In all regions, the prevalence of having had an STI increased with age until the early/mid 30s, after which it stabilized or declined (Fig. 2D). The adjusted odds for ever having had an STI was significantly higher in the north (OR (95% CI): 1.24 (1.13 to 1.36)), significantly lower in the west (0.89 (0.83 to 0.97)), and similar to the national average in the middle region (0.93 (0.85 to 1.02)) as well as in the southeast (0.97 (0.91 to 1.03)).

Current smoking was reported by 33.7% of Norwegian women aged 18–45 in 2005. The current smoking prevalence reported in the north, middle, southeast and west, were 38.0, 32.3, 33.4 and 33.4%, respectively. In age-adjusted logistic regression analyses, the current smoking prevalence in the southeastern region and the western region did not differ from the national level (OR (95% CI): 0.96 (0.91 to 1.01), and 0.96 (0.90 to 1.02), respectively), while the middle region had slightly lower odds (0.92 (0.85 to 0.99)), and the northern region had higher odds for current smoking (1.18 (1.09 to 1.28)).

Ever having used hormonal contraceptives was reported by 87.5% of Norwegian women aged 18–45 in 2005, with similar proportions in each region (89.0, 88.6, 87.3 and 86.8% in the north, middle, southeast and west, respectively). In adjusted logistic regression analyses, ever-use of hormonal contraceptives did not differ significantly from the national level in any of the regions (north: 0.87 (0.76 to 1.01); middle: 1.07 (0.93 to 1.22); southeast (0.98 (0.90 to 1.07); west (1.10 (0.98 to 1.23)).

The screening coverage rate was on average 69.6% in Norway as a whole during the period 1995 to 2016, and each of the four regions differed by less than one percentage point from this national estimate. The slight changes in screening coverage observed over the study period were similar in the four regions (Fig. 2E).

## Discussion

We observed some regional differences in cervical cancer incidence rates, as well as in incidence trends over the period investigated. The highest incidence rates were consistently observed in the northern and southeastern regions, where we also observed a significant decrease in cervical cancer incidence over time. In contrast, the western and middle regions had relatively lower incidence rates that did not change significantly during the 1992–2016 study period. The regional differences in incidence rates were particularly high during 1997–2006 and diminished thereafter. We also observed some consistent regional differences in behaviors associated with risk of cervical cancer. Women in the northern region on average reported the earliest sexual debut, and the highest proportion of debut before age of consent, number of sexual partners, prevalence of ever having had an STI and prevalence of current smoking. In contrast, women in the western region on average reported the latest sexual debut, and the lowest proportion of debut before legal age of consent, number of sexual partners and prevalence of ever having had an STI.

The northern region ranks high for cervical cancer incidence rates as well as for estimates of risk behaviors for cervical cancer, while the western region ranks low for either type of endpoint. The southeastern region ranks high for cervical cancer incidence rates and intermediate for risk behaviors, and the middle region ranks low for cervical cancer incidence rates and intermediate for behavioral risk factors. Hence, to some extent there is concordance between regional patterns in cervical cancer incidence and its main behavioral risk factors. However, the analyses also show that the concordance is not complete, indicating that other factors also may influence the observed regional differences in cervical cancer incidence. The background risk for HPV exposure is associated with social norms that probably will show broadly similar trends across regions and will not change abruptly. However, this is not what we observe for the overall cervical cancer and the squamous cell carcinoma incidence rate trends. They are stable in some regions and decrease in others, and even go in opposite directions within the study period in two of the four regions. Squamous cell carcinoma may be prevented by screening [1], and there are several performance parameters that affect screening efficiency [26]. The screening coverage rate is an important factor, but coverage did not differ much between regions during the study period and hence does not appear to be an important determinant for the regional differences in cervical cancer incidence observed in the present study. Other screening performance parameters include test sensitivity, both in terms of the type of test used and in terms of the performance and interpretation of these tests, and the management of abnormalities. These factors could, in addition to differences in background risk, be associated with the observed regional differences in cervical cancer rates. In Norway, the screening guidelines and the screening program as a whole are national, but the present study indicates that the clinical screening performance to some extent still may differ between regions.

In contrast to the squamous cell carcinoma incidence trends, the adenocarcinoma trends are similar between regions and do not change abruptly. They may better reflect trends in background exposure to HPV since adenocarcinomas are not efficiently prevented by cytological screening [3, 27]. We have previously documented that there have been modest secular trends towards lower debut ages and more sexual partners among Scandinavian women during recent decades [28], which is consistent with the adenocarcinoma trends documented in the present study. A similar pattern for age at sexual debut is indicated here in each region, with a decrease in debut age with decreasing age at survey response, and a corresponding increasing frequency of debut before legal age of consent.

Routine school-based HPV vaccination was started in Norway in 2009, with girls born 1997 as the oldest eligible birth cohort. For the duration of the present study, there was no catch-up vaccination of older birth cohorts and negligible opportunistic vaccination [29]. Thus, HPV vaccination is not likely to have had any effect on the cancer rates reported here. HPV prevalence among women at screening-age in Norway has so far been found to be stable in pre- and post-vaccination era surveys [29], while considerably lower HPV prevalence has been observed in vaccinated than in unvaccinated birth cohorts below screening age [30].

A strength of the present study is the nationally complete cancer incidence data [16], which minimizes the risk of information and selection bias. Moreover, the population-based design and the relatively high survey response rate and small sociodemographic differences between responders and non-responders indicate that the survey data are generalizable to the Norwegian female population [28, 31]. However, large surveys always have non-responders and are thus prone to selection bias. The main limitation of the study is that its design does not permit causal inference regarding the observed regional differences in cervical cancer incidence and risk behavior. However, the association between cervical cancer risk and the behaviors addressed here have previously been firmly established [8–10]. Further limitations are that the survey data was collected only once (in 2005), that survey data are prone to information bias, and that there may be residual confounding in the survey analyses. Finally, we lack data on reproductive factors, which may exert independent effects on cervical cancer risk. However, such effects are prominent only for women with a high number of births [11], which is uncommon in developed countries like Norway. Since the limitations do not or are unlikely to differ by region, we do not expect they exert a great effect on the regional survey comparisons.

Organized recommendations for cervical screening are still largely the same for all women. However, as this study indicates, the risk for cervical cancer is not, because it is influenced by individual factors such as risk-taking and preventive health behavior. More personalized cervical cancer screening strategies will most likely be inevitable when birth cohorts with a high HPV vaccine uptake reach screening age, because the risk of HPV-related cervical disease is strongly reduced by HPV vaccination [32]. Also taking additional individual risk indicators into account, such as risk-taking behavior and previous screening history, could improve cervical screening further and potentially reduce the regional differences in cervical cancer incidence observed in the present study.

We conclude that there were regional differences in cervical cancer incidence rates throughout the duration of the nationwide organized cervical screening program in Norway, and that the differences have decreased in recent years. Moreover, there were regional differences in behaviors associated with cervical cancer risk that to some extent corresponded to the observed differences in incidence rates, while screening coverage rates were similar between regions. Regional differences in risk behaviors may in part explain the regional differences in cervical cancer incidence.

## Supplementary Information

**Additional file 1.**

## Data Availability

The registry data can be accessed for relevant research purposes by application to the CRN. The survey data are not publicly available because they are detailed and sensitive and the participants have not consented to such transfer of data. Access to a completely anonymized version of parts of the survey data can be granted upon reasonable request to the corresponding author.

## Abbreviations

HPV: human papillomavirus
APC: annual percentage change
AAPC: average annual percentage change
HR: hazard ratio
OR: odds ratio
IQR: interquartile range
SE: standard error
N: sample size
Adj: adjusted
STI: sexually transmitted infection
SCC: squamous cell carcinoma
ADC: adenocarcinoma
CI: confidence interval
NCCSP: Norwegian Cervical Cancer Screening Program
CRN: Cancer Registry of Norway
