# Supplementary material for: Regional differences in cervical cancer incidence and associated risk behaviors among Norwegian women: a population-based study
Source: BMC Cancer. 2021 Aug 19;21:935. doi: 10.1186/s12885-021-08614-w (PMC8377942; doi:10.1186/s12885-021-08614-w)
Supplement: Supplementary file 1 — Additional file 1. [file 12885_2021_8614_MOESM1_ESM.docx]

| **Supplementary table 1. Sociodemographic characteristics among women by health region in Norway in 2005^1^** | | | | |
| --- | --- | --- | --- | --- |
|  | **Southeast** | **West** | **Mid** | **North** |
| *Total female population (N)* | 1293792 | 474861 | 323508 | 230132 |
| *Proportion by age group (%)* |  |  |  |  |
| 0-9 | 12.1 | 13.7 | 12.6 | 12.5 |
| 10-19 | 12.0 | 13.6 | 13.2 | 13.2 |
| 20-29 | 12.0 | 12.7 | 11.8 | 11.3 |
| 30-39 | 15.2 | 14.5 | 13.8 | 14.3 |
| 40-49 | 13.9 | 13.3 | 13.3 | 13.7 |
| 50-59 | 12.9 | 11.9 | 12.9 | 12.9 |
| 60-69 | 8.8 | 8.0 | 8.7 | 9.0 |
| 70-79 | 7.1 | 6.5 | 7.4 | 7.3 |
| 80+ | 6.1 | 5.8 | 6.4 | 5.9 |
|  |  |  |  |  |
| *Proportion of immigrants^2^ (%)* |  |  |  |  |
|  | 8.3 | 5.1 | 3.9 | 4.9 |
|  |  |  |  |  |
| *Proportion by education level^3^ (%)* |  |  |  |  |
| None/unknown^4^ | 2.5 | 1.8 | 1.4 | 1.5 |
| Primary | 32.4 | 32.4 | 33.6 | 39.0 |
| Secondary | 39.2 | 40.6 | 41.3 | 36.8 |
| University | 25.9 | 25.2 | 23.7 | 22.7 |

^1^ Source: <https://www.ssb.no/statbank/>

^2^ Women with parents and grandparents who were not born in Norway

^3^ Among women age 16 or older
